# Efficacy of Camrelizumab in Advanced Non-Small-Cell Lung Cancer and Prognostic Analysis of Different PET/CT Features

**DOI:** 10.1155/2022/9942918

**Published:** 2022-03-25

**Authors:** Jinhai Zou, Chunying Li, Yu Han, Yu Xing, Yingying Zhan, Chao Zuo, Xinyue Ren, Rongge Xing, Nan Zhang

**Affiliations:** ^1^Department of Nuclear Medicine, Cangzhou Central Hospital, Cangzhou, China; ^2^Department of Digestive Medicine, Cangzhou Central Hospital, Cangzhou, China; ^3^Department of Pathology, Cangzhou Central Hospital, Cangzhou, China; ^4^Department of Cardiothoracic Surgery, Cangzhou Central Hospital, Cangzhou, China

## Abstract

**Objective:**

To evaluate the efficacy of the PD-1 inhibitor camrelizumab plus chemotherapy in the first-line treatment of advanced non-small-cell lung cancer (NSCLC) and the prognostic differences of patients with different PET/CT features.

**Methods:**

Between December 2018 and October 2020, 100 patients with NSCLC assessed for eligibility treated in our institution were recruited and randomly assigned (1 : 1) to receive either the TC regimen chemotherapy (control group) or the TC regimen chemotherapy plus camrelizumab (study group). The primary endpoints were clinical efficacy, progression-free survival (PFS), and overall survival (OS). A decrease of max standard uptake value (SUVmax) of >30% in primary lung cancer was considered as metabolic remission. The prognostic differences of the eligible patients with different PET/CT features were assessed. Survival data were analyzed using the Kaplan-Meier method to obtain the survival rate and calculate the median survival time.

**Results:**

The metabolic remission rate and objective remission rate were significantly higher with chemotherapy plus camrelizumab versus chemotherapy alone. The study group had significantly higher CD3+ and CD4+ T-cell ratios and CD4+/CD8+ ratio and significantly lower CD8+ T-cell ratio than the control group after treatment. PFS (10 months versus 4 months) and OS (HR = 37.094, *P* ≤ 0.001) were better with camrelizumab plus chemotherapy versus stand-alone chemotherapy. The incidence of adverse events (AE) was similar between the two groups. The patients in the study group were stratified into metabolic remission and metabolic nonremission based on PET/CT results. Intersubgroup analysis showed significantly better PFS and OS in the metabolic remission group than in the nonmetabolic remission group.

**Conclusion:**

The camrelizumab plus chemotherapy as a first-line treatment option for NSCLC significantly increases the survival benefit. Metabolic status shown by PET/CT correlates with long-term prognosis and demonstrates a great potential for early assessment of efficacy to support the choice of treatment regimens.

## 1. Introduction

Lung cancer is the most common malignancy and is the number one cause of cancer death [1]. Non-small-cell lung cancer (NSCLC) is a common type of pulmonary carcinoma, including squamous cell carcinoma (squamous carcinoma), adenocarcinoma, and large cell carcinoma. The clinical manifestations of NSCLC are chest distention and pain, bloody sputum, low fever, cough, fatigue, weight loss, loss of appetite, dyspnea, and hemoptysis, which are associated with about 80% of all lung cancers [2]. Due to the atypical early symptoms of NSCLC, approximately 75% of lesions are already in the advanced stage at the time of diagnosis with poor 5-year survival [3]. Currently, an urgent need for effective therapies for advanced NSCLC exists due to the limited remission rate and survival extension with standard chemotherapy and the inevitability of drug resistance after long-term use [4].

Programmed cell death protein (PD-1) is a protein preferentially expressed in the body's immune B, T, and NK cells, involved in antigen presentation and expressed by multiple cell types, providing a new direction for NSCLC immunotherapy [5]. PD-1 inhibitor therapy relies on biomarkers other than the source of the tumor and has a broader spectrum of anticancer effects [6]. PD-1 inhibitors allow long-term survival and even clinical cure of patients with advanced tumors with no predisposition to drug resistance, which significantly outperforms other treatment modalities. In addition, PD-1 inhibitor treatment activates the immune system of the body to attack the tumor with much fewer overall side effects [7]. PD-1 inhibitors have been officially approved by the US FDA since 2014 for malignant melanoma, non-small-cell lung cancer, liver cancer, gastric cancer, kidney cancer, bladder cancer, head and neck tumors, Hodgkin's lymphoma, Merkel cell carcinoma, and all solid tumors with high microsatellite instability [8]. PD-1 inhibitors could serve as one of the treatment options for NSCLCs with an EGFR mutation and high PD-L1 expression [9]. PD-1 inhibitors in combination with chemotherapy have been approved for the first-line treatment of advanced non-small-cell lung cancer [9, 10]. However, the efficiency of PD-1 inhibitors in unselected patients with solid tumors only ranges from 10% to 30%. Commonly used methods for patient screening include determination of PD-L1 expression, MSI assay, tumor mutational load (TMB) assay, and tumor-infiltrating lymphocyte (TIL) assay; however, their operations are complicated with a low accuracy [11]. Imaging alterations are the main means to assess the effectiveness of tumor treatment. ^18^F-fluorodeoxyglucose (^18^F-FDG) positron emission tomography/computed tomography (PET/CT) effectively reflects the metabolism of the lesion and assesses the effect of early radiotherapy in lung cancer patients [12]. In the present study, the PD-1 inhibitor camrelizumab was used in combination with chemotherapy as the first-line treatment modality for NSCLC, and ^18^F-FDG PET/CT was used to assess patient outcomes early after treatment to evaluate its consistency with long-term outcomes and to provide a basis for the selection of patient treatment modality.

## 2. Materials and Methods

### 2.1. Study Design

Between December 2018 and October 2020, 100 patients with NSCLC assessed for eligibility treated in our institution were recruited and randomly assigned (1 : 1) to either the control group (*n* = 50) or the study group (*n* = 50). All patients were assessed for clinical efficacy using CT at 6 months after treatment, and the study group was assessed for tumor metabolic status using ^18^F-FDG PET/CT at 1 month after the intervention to compare the concordance with CT results. All patients had signed the informed consents. The study protocol was approved by the medical ethnic review board.

### 2.2. Inclusion and Exclusion Criteria

#### 2.2.1. Inclusion Criteria

Inclusion criteria are as follows: (1) patients aged 36-94 years; (2) with histologically or cytologically confirmed advanced (stage IIIB/IIIC/IV), squamous or nonsquamous NSCLC; (3) with Eastern Cooperative Oncology Group (ECOG) [13] score of 1 to 3 points; (4) with an expected survival of >3 months; and (5) with measurable lesions by Response Evaluation Criteria in Solid Tumors (RECIST) criteria [14].

#### 2.2.2. Exclusion Criteria

Exclusion criteria are as follows: (1) patients with mixed malignancy; (2) with primary malignancy at other sites; (3) with severe organ dysfunction that cannot tolerate treatment; (4) with other antitumor therapy or previous immunotherapy; and (5) patients who cannot comply with the trial protocol or cannot cooperate with the follow-up.

### 2.3. Detection Methods

#### 2.3.1. ^18^F-FDG PET/CT Imaging

A GE Discovery STE 16 PET/CT scanner was used with ^18^F-FDG as the imaging agent. Before the examination, the patients were fasted for more than 6 h with the blood glucose level maintained between 4.5 and 7.8 mmoL/L, received an intravenous injection of ^18^F-FDG 3.7~4.44 MBq/kg, and laid down at rest for 45~60 min, followed by the examination after urination. The CT scan was performed covering a range from the skull to the thigh, with the scan parameters being voltage of 120 kV, current of 150 mA, and layer thickness of 3.75 mm. PET scans were performed in 3D for 3 min per bed, with a layer thickness of 3.27 mm, and the images were obtained after attenuation correction and iterative method reconstruction and transmitted to Xileris workstation for fusion reconstruction.

#### 2.3.2. CT Scan

The CT scan voltage was set to 120 kV, current to 180 mA, pitch to 3.75 : 1, spiral time to 0.8 s/week, bed speed to 22.5 mm/s, and matrix to 512 × 512.

### 2.4. Treatment Methods

The control group was treated with the TC chemotherapy regimen, i.e., paclitaxel and carboplatin. Paclitaxel (manufacturer: Yangtze River Pharmaceutical Group Co., Ltd.; approval number: State Drug quantification: H20053001): 135 mg/m^2^, added to 0.9% sodium chloride injection 500 mL, intravenous drip for >3 h, d 1. Carboplatin (manufacturer: Qilu Pharmaceutical Co., Ltd.; approval number: State Drug quantification: H20020181): 20 mg/m^2^, added to 5% glucose injection 500 mL, titrated intravenously for 2 h, d 1-4. One course of treatment spanned 21 days. On top of the TC chemotherapy regimen, the study group was given additional camrelizumab (manufacturer: Yangtze River Pharmaceutical Group Co., Ltd; approval number: State Drug quantification: H20053001) at a dose of 200 mg/time, and the drip duration was maintained at 30-60 min. Before the intravenous drip, 200 mg of camrelizumab was redissolved with 5 mL of sterilized water for injection and then diluted with 100 mL of 5% glucose injection, which was started 30 min after the cisplatin drip. One course spanned 21 days, and the administration was performed on the first day of each course. All eligible patients were given three consecutive courses.

### 2.5. Outcomes

#### 2.5.1. Clinical Efficacy

After 1 course of treatment, ^18^F-FDG PET/CT imaging was performed, and ^18^F-FDG PET/CT uptake was evaluated by visual inspection and semiquantitative analysis. A decrease of max standard uptake value (SUVmax) of >30% in primary lung cancer was considered as metabolic remission, and a decrease of ≤30% was considered as metabolic no remission. CT examinations were performed after 3 courses of treatment, and clinical outcomes were classified as complete remission (CR), partial remission (PR), stable disease (SD), and progressive disease (PD) as per RECIST criteria [14]. CR: tumor lesions disappeared; PR: tumor lesions decreased by less than 25-50% of the product of two diameters and no new lesions occurred; SD: tumor lesions decreased by less than 25% of the product of two diameters and increased by less than 25%; PD: tumor lesions increased by more than 25% of the product of two diameters and new lesions occurred. Objective remission rate (ORR) = (CR + PR)/total number of cases × 100%.

#### 2.5.2. T-Cell Subsets and NK Cell Ratios

Before treatment and after 3 courses of treatment, flow cytometry was used to determine the changes in the ratio of CD3+, CD4+, and CD8+ T-cell subsets in the peripheral blood of patients. The reagents used were provided by ThermoFisher, and the instructions were strictly followed.

#### 2.5.3. Long-Term Outcome

After treatment, all participants were followed up once a month, and overall survival (OS) and progression-free survival (PFS) were recorded as per RECIST criteria [14]. OS was recorded from the time point of chemotherapy to death from any cause. PFS was recorded from the time point of the chemotherapy to the first disease progression or death.

#### 2.5.4. Quality of Life

The Karnofsky performance scale (KPS) [15] was used to assess the quality of patients after treatment. The scale is scored 100 points, and the higher the score, the better the health status, and the more tolerable the side effects of treatment. A KPS score of ≥80 points or more was considered independent, i.e., self-care; 50-70 points were considered semidependent, i.e., semiself-care; less than 50 points were considered dependent, i.e., requiring assistance in daily life.

#### 2.5.5. Adverse Events

Adverse events (AEs) such as rash, gastrointestinal reactions, a small amount of coughing blood, and bone marrow suppression during the follow-up period were recorded. In the case of 2 or more concurrent toxic side effects in the same patient, the most serious toxic side effect was counted.

### 2.6. Statistical Analysis

SPSS 23.0 was used for data collation and analyses, and GraphPad Prism 9.0 was used to plot the graphics. The measurement data were expressed as (^−^*x* ± *s*), and differences between groups were examined by *t*-test. The count data were expressed as rate (%), and differences between groups were examined using the chi-square test. Survival data were analyzed using the Kaplan-Meier method to obtain the survival rate, calculate the median survival time, and describe the survival process through survival curves. The difference was verified at *α* = 0.05 as the threshold of significance.

## 3. Results

### 3.1. Baseline Features

The two groups of patients showed similar clinical baseline features such as age, gender, course of disease, and underlying disease (*P* > 0.05) (Table 1).

### 3.2. Metabolic Remission

The control group had 17 cases of metabolic remission and 33 cases of metabolic nonremission, with a metabolic remission rate of 34%. The study group had 28 cases of metabolic remission and 22 cases of metabolic nonremission, with a metabolic remission rate of 56%. The eligible patients given camrelizumab showed better metabolism benefits versus those given chemotherapy only (*P* = 0.027). (Table 2).

### 3.3. Clinical Efficacy

After three courses of treatment, the control group had 15 cases of PR, 14 cases of SD, and 21 cases of PD, with an ORR of 30% (15/50). The study group had 19 cases of PR, 20 cases of SD, and 11 cases of PD, with an ORR of 38% (19/50). The ORR was significantly higher with chemotherapy plus camrelizumab versus with chemotherapy alone (*P* = 0.032) (Table 3).

### 3.4. Immunologic Function

Before treatment, the two groups showed similar ratios of CD3+, CD4+, CD8+ T cells, and CD4+/CD8+ ratio. The study group had significantly higher CD3+ and CD4+ T-cell ratios and CD4+/CD8+ ratio and significantly lower CD8+ T-cell ratio than the control group after treatment (*P* < 0.05) (Table 4).

### 3.5. Long-Term Outcome

**Figure 1:**
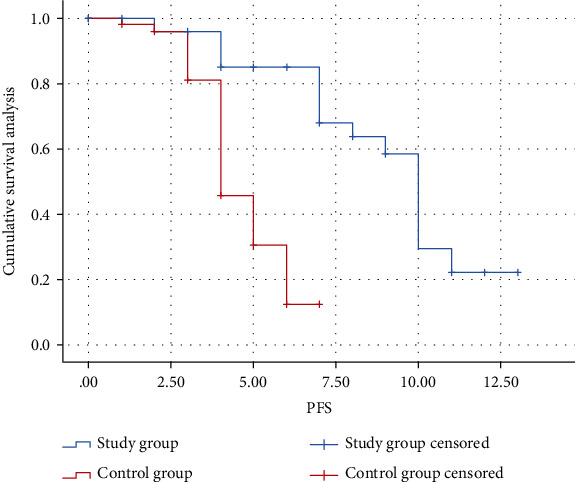
Kaplan-Meier estimates of PFS.

**Figure 2:**
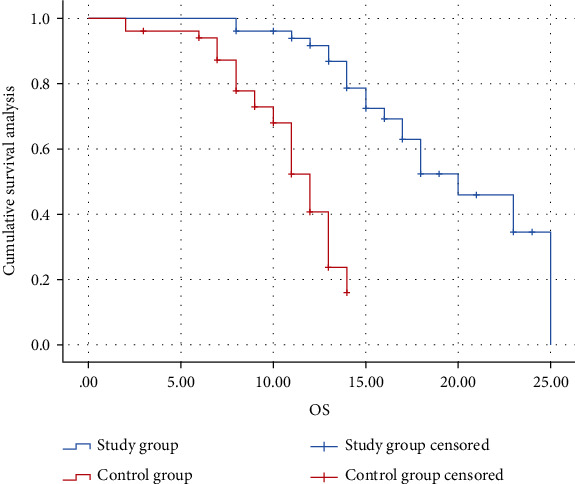
Kaplan-Meier estimates of OS.

The median PFS was 10 months (95% CI: 9.244 to 10.756) in the study group and 4 months (95% CI: 3.396 to 4.604) in the control group (HR = 30.862, *P* ≤ 0.001). The median OS in the control group was 12 months (95% CI: 10.854 to 13.146). The study group outperformed the control group in terms of the median OS (HR = 37.094, *P* ≤ 0.001) (Figures 1 and 2).

### 3.6. Prognosis of Different PET/CT Features

**Figure 3:**
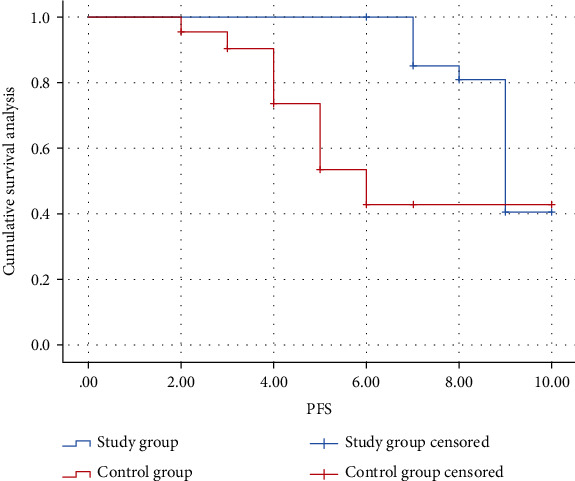
Kaplan-Meier estimates of PFS of subgroups in study group.

**Figure 4:**
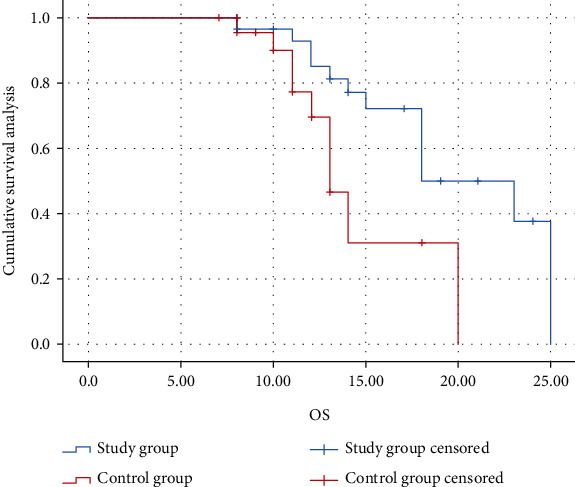
Kaplan-Meier estimates of OS of subgroups in study group.

The median PFS was 4.5 months (95% CI: 4.254-7.755) and 8.5 months (95% CI: 8.476-9.524) in the nonremission and remission groups, and median OS was absent in both subgroups. The Kaplan-Meier analysis showed that both PFS and OS were significantly better in the metabolic remission group than in the nonremission group (*P* < 0.05) (Figures 3 and 4).

### 3.7. Adverse Event

There were 37 cases of AEs in the control group, including 5 cases with grade 3 AE or higher, and there were 41 cases of AEs in the study group, including 7 cases with grade 3 AE or higher (*P* > 0.05) (Table 5).

## 4. Discussion

In the present study, the PD-1 inhibitor camrelizumab plus chemotherapy obtained better clinical benefits as a first-line treatment for NSCLC by significantly prolonging the PFS and OS, with a high safety profile. Most NSCLC cases are diagnosed at advanced stages due to their rapid progression resulting in the ineligibility for surgery. The current status of survival and prognosis of advanced NSCLC is unsatisfactory due to the unavailability of effective interventions [16]. PD-1 inhibitors are immune checkpoint inhibitors that exhibit antitumor efficacy and safety in multiple solid tumors, providing a new direction for immunotherapy in advanced NSCLC. The Keynote-021 study confirmed that PD-1 inhibitors significantly prolonged the primary observational endpoint PFS (13 months vs. 8.9 months) and elevated ORR (55% vs. 29%) compared to standard first-line chemotherapy [17]. Alwithenani et al. used camrelizumab in combination with carboplatin + pemetrexed for the first-line treatment of advanced/metastatic EGFR/ALK mutation-negative nonsquamous non-small-cell lung cancer, which significantly prolonged the PFS and reduced the risk of death by 27% [18]. Herein, the joint use of camrelizumab with TC chemotherapy regimen significantly enhanced ORR, improved immune function, and prolonged PFS and OS, confirming a significant survival benefit of camrelizumab over conventional chemotherapy regardless of PD-L1 expression status and histological staging, which was consistent with the results of the previous study. Moreover, the addition of camrelizumab did not elicit other adverse events, which exhibits a high safety profile.

Research has shown that the efficacy of PD-1 inhibitors is subject to the pathological staging of lung cancer and PD-L1 expression, and the higher the PD-L1 expression, the better the effect of PD-1 inhibitors. Thus, PD-L1 expression assays serve as complementary diagnostics to assist in screening populations for potential benefits from immunotherapy [19]. Nevertheless, the determination of PD-L1 expression in clinical applications is complicated and less accurate. Thus, prediction of clinical efficacy at an early stage to select a more appropriate drug regimen is a current pressing issue to be addressed. CT morphological changes are recommended to assess the clinical efficacy of tumors, but the late morphological changes of lesions result in poor accuracy in the assessment of early outcomes [20]. ^18^F-FDG PET/CT imaging combines two imaging methods, PET and CT, to achieve simultaneous structural and functional imaging, effectively reflecting the metabolic situation of the lesion and assessing the early tumor efficacy. Lung cancer tissues proliferate with a substantial increase in nutritional requirements and metabolic levels, with the massive uptake of ^18^F-FDG for conversion to 6-phospho-FDG, which remain in the cells and are easily visualized, enabling earlier determination of clinical outcome versus morphological changes [21]. In the present study, both PFS and OS were significantly better in the metabolic remission group than the metabolic nonremission group, confirming the consistency of PET/CT with long-term prognosis.

To sum up, the PD-1 inhibitor camrelizumab plus chemotherapy significantly increased survival benefit and improved immune function in advanced NSCLC with a promising safety profile. Assessment of tumor metabolic status using PET/CT is concordant with long-term prognosis and demonstrates great potential as an early assessment of treatment efficacy to provide a reference for the selection of future treatment regimens.

## Figures and Tables

**Table 1:** Clinical baseline features.

	Control group (*n* = 50)	Study group (*n* = 50)	*t*/*χ*^2^	*P*
Age ($\bar{x}\pm \text{s}$, years)	67.12 ± 12.41	65.82 ± 14.02	0.491	0.625
Gender (*n*)			1.169	0.280
Male	32	37		
Female	18	13		
Smoking (*n*)			0.361	0.548
Yes	25	22		
No	25	28		
Histology (*n*)			0.480	0.488
Squamous	11	14		
Nonsquamous	39	36		
Brain metastasis (*n*)			0.832	0.362
Yes	15	11		
No	35	39		
Stage (*n*)			0.679	0.410
III	17	21		
IV	33	29		
ECOG score (*n*)			0.683	0.711
1	9	7		
2	24	28		
3	17	15		

ECOG: Eastern Cooperative Oncology Group.

**Table 2:** Metabolic remission by PET/CT.

	Metabolic remission	Metabolic nonremission
Control group (*n* = 50)	17	33
Study group (*n* = 50)	28	22
*χ* ^2^	4.889	
*P*	0.027	

**Table 3:** Clinical efficacy.

	CR	PR	SD	PD	ORR
Control group (*n* = 50)	0	15	14	21	15
Study group (*n* = 50)	0	19	20	11	19
*χ* ^2^					4.596
*P*					0.032

CR: complete remission; PR: partial remission; SD: stable disease; PD: progressive disease; ORR: objective remission rate.

**Table 4:** Immunologic function.

		CD3+	CD4+	CD8+	CD4+/CD8+
Control group (*n* = 50)	Before	35.51 ± 5.03	38.17 ± 4.64	37.52 ± 4.26	0.91 ± 0.27
	After	25.25 ± 5.26 a	26.75 ± 4.15 a	50.26 ± 5.26 a	0.56 ± 0.16 a
Study group (*n* = 50)	Before	34.13 ± 4.82	37.82 ± 4.75	38.92 ± 4.47	0.92 ± 0.22
	After	28.39 ± 4.16 ab	31.15 ± 3.53 ab	44.17 ± 4.02 ab	0.69 ± 0.14 ab

Note: a indicated *P* < 0.05 compared with before treatment and b indicated *P* < 0.05 compared with the control group.

**Table 5:** Adverse events.

	All grades	Grades ≥ 3
Control group (*n* = 50)	37	5
Study group (*n* = 50)	41	7
*χ* ^2^	0.932	0.379
*P*	0.334	0.538

## Data Availability

The datasets used during the present study are available from the corresponding author upon reasonable request.
